# Towards Human-Like Emergent Communication via Utility, Informativeness, and Complexity

**DOI:** 10.1162/opmi_a_00188

**Published:** 2025-04-02

**Authors:** Mycal Tucker, Julie Shah, Roger Levy, Noga Zaslavsky

**Affiliations:** Anthropic; Department of Aeronautics and Astronautics, Massachusetts Institute of Technology; Department of Brain & Cognitive Sciences, Massachusetts Institute of Technology; Department of Psychology, New York University

**Keywords:** information theory, compression, emergent communication, categorization

## Abstract

Two prominent, yet contrasting, theoretical views are available to characterize the underlying drivers of language evolution: on the one hand, task-specific utility maximization; on the other hand, task-agnostic communicative efficiency. The latter has recently been grounded in an information-theoretic tradeoff between communicative complexity and informativeness, known as the Information Bottleneck (IB) principle. Here, we integrate these two views and propose an information-constrained emergent communication framework that trades off utility, informativeness, and complexity. To train agents within our framework, we develop a method, called Vector-Quantized Variational Information Bottleneck (VQ-VIB), that allows agents to interact using information-constrained discrete communication embedded in a continuous vector space. We test this approach in three domains and show that pressure for informativeness facilitates faster learning and better generalization to novel domains. At the same time, limiting complexity yields better alignment with actual human languages. Lastly, we find that VQ-VIB outperforms previously proposed emergent communication methods; we posit that this is due to the semantically-meaningful communication embedding space that VQ-VIB affords. Overall, our work demonstrates the role of cognitively-motivated optimality principles in inducing aspects of human-like communication among artificial agents.

## INTRODUCTION

What computational principles underlie language evolution? One common answer to this question stems from game-theoretic approaches of language evolution that emphasize utility maximization (Chaabouni, Strub, et al., 2021; Steels & Belpaeme, 2005; Still & Precup, 2012). In this view, agents learn to interact in a given environment in order to accumulate maximal expected utility, or reward, in a cooperative task. An alternative answer, which has become prominent in the cognitive linguistic literature, is that language is shaped by a general pressure for efficient communication (for review see Gibson et al., 2019; Kemp et al., 2018). Most relevant to our work is a recent line of work that argues that human language is characterized by near-optimal compression of meanings into words (Zaslavsky, 2020; Zaslavsky et al., 2018). Zaslavsky et al. (2018) formulated this compression mechanism in human languages using the information bottleneck (IB) optimality principle (Tishby et al., 1999), which can be interpreted as a tradeoff between communicative informativeness (roughly, how well a listener can understand a speaker, regardless of any specific context or task) and communicative complexity (roughly, how many bits are allocated for communication). These two frameworks for communication—utility maximization and communicative efficiency—represent different hypotheses about the underlying factors that may drive language evolution.

**Figure 1.**
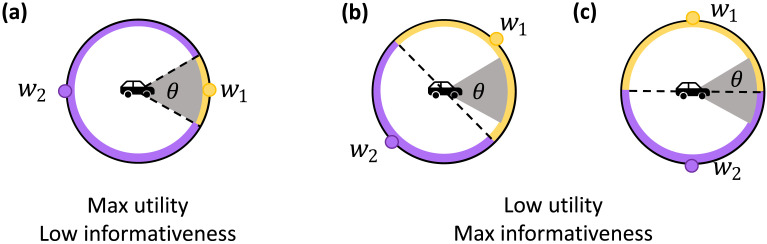
In our driving example, a passenger wishes to communicate with a driver about driving straight. High utility is achieved if the steering wheel’s angle is within the gray region. Focusing on systems with two words (yellow angles map to word *w*_1_, purple angles map to word *w*_2_) illustrates the tension between maximizing utility and maximizing informativeness. (a) Utility-maximizing communication divides angles according to whether they are within the gray region. As *θ* → 0, this will yield non-informative communication that does not generalize to other tasks. (b–c) Maximally informative communication uses the two signals to divide the circle by half, yielding maximal reconstruction accuracy of the angle. However, this strategy does not distinguish between a system with sub-optimal but still non-trivial utility (b) and a system that leads to the worst-case utility in this task (c).

While utility and informativeness may seem similar, we emphasize that these are distinct concepts that can induce competing pressures. Utility is a *task-specific* measure that captures external (non-communicative) goals in the world, while informativeness is a *task-agnostic* measure that captures the communicative goal of conveying meanings accurately, regardless of any specific downstream task. To see how these two concepts differ, consider a driving scenario (Figure 1) where a passenger communicates with a driver about steering a car. High utility is attained if the driver orients the steering wheel within a range of angles that roughly correspond to driving straight; however, the driver is inattentive and must be reminded by a passenger of the actual angle of the car. Even in highly simplified communication systems with only two words, passengers and drivers may derive different communication strategies depending upon the relative importance of utility (keep the steering wheel within the acceptable angle range) and informativeness (allow the driver to infer the intended angle of the steering wheel). For example, utility-based communication will divide the angles into two regions corresponding to “straight” (within the gray area in Figure 1) and “veering” (Figure 1a). Conversely, informativeness-based communication will divide the circle by half, regardless of which angles are considered acceptable (Figure 1b–c), which leads to worse utility in this case but could support high performance in other tasks (e.g., turn left or right). As the acceptable range of driving angles shrinks, the distinction between utility-optimal and informativeness-optimal communication will continue to grow, approaching an extreme of non-informative but maximal-utility communication, which will not generalize across tasks.

This example demonstrates the importance of balancing task-specific utility and task-agnostic communicative efficiency. However, the interaction between these factors has been largely under-explored in the literature, leaving open three key questions that we aim to address in this work.

First, it is unclear what kind of evolutionary learning dynamics may allow interlocutors to optimally balance utility, complexity, and informativeness, and to what extent such dynamics may explain human semantic systems (i.e., mappings from meanings to words). While utility-based approaches are typically formulated with respect to specific learning dynamics, efficient communication approaches, including IB, are typically not (Levinson, 2012). Instead, they focus on population-level characteristics of language that are either stationary (e.g., Regier et al., 2015) or linked to dynamical processes that are not explicitly grounded in agent interactions, such as annealing (Zaslavsky, 2020) or semantic chaining (Y. Xu et al., 2016). At the same time, the IB framework for semantic systems offers a compelling theoretical explanation for several empirical phenomena, including cross-language semantic variation, efficiency of graded categories and inconsistent naming, and the continuous refinement (or coarsening) of semantic systems as they evolve. Utility-based approaches, including recent studies that evaluate the communicative efficiency of utility-maximizing systems (e.g., Carlsson et al., 2023; Chaabouni, Kharitonov, et al., 2021; Kågebäck et al., 2020) lack this type of explanatory power and instead turn to assumptions about environmental factors (e.g., noise or external rewards) and fixed vocabulary sizes to account for cross-language variation. Thus, it is an open question how languages may evolve by simultaneously balancing utility-based and IB-based pressures.

Second, while utility-based approaches have been applied to open domains, where the set of possible referents is not restricted to a predefined set, they struggle to generalize to novel out-of-distribution inputs (Chaabouni, Strub, et al., 2021). Intuitively, pressure for task-agnostic communicative informativeness could facilitate better generalization. However, efficient communication approaches have so far focused on closed domains (Kemp et al., 2018), and it has not been clear how to extend this approach to open domains. Thus, an important open question is to what extent the interplay between utility and IB may be beneficial for open-domain communication.

Finally, both utility-based and efficiency-based approaches to language evolution have primarily focused so far on simple reference games. While some studies in the machine-learning emergent communication literature have focused on other reinforcement learning settings (Foerster et al., 2016; Mordatch & Abbeel, 2018, e.g.,), they have not made direct contact with general, cognitively-motivate principles that are believed to underlie human language, such as IB. Therefore, it remains unclear whether the integration of utility and IB optimization may also apply to more complex tasks such as navigation, that require multi-agent communication.

In this work, we propose a framework that integrates the utility-based and IB-based approaches to language evolution, which we call information-constrained emergent communication (ICEC), and show how this framework can address the three aforementioned open questions. The key component of our framework is a novel deep-learning method we develop, which enables artificial agents to communicate using discrete symbols that are embedded in a continuous vector space, much like word embeddings (Pennington et al., 2014), while being guided by pressure to optimally trade off utility, informativeness, and complexity. We refer to this method as VQ-VIB because it combines ideas from variational approximations of IB (VIB, Alemi et al., 2017) and vector quantization (VQ) for discrete representation learning (van den Oord et al., 2017). We evaluate VQ-VIB in three settings that, respectively, address the three open questions discussed above: (1) color reference game, showing that the evolutionary learning dynamics of VQ-VIB agents can yield emergent color naming systems that are human-like; (2) open-domain communication about objects in naturalistic images, showing that taking into account informativeness facilitates out-of-domain generalization, and taking into account complexity facilitates alignment between artificial agents and English speakers; and (3) navigation task, demonstrating the applicability of our approach beyond simple reference games. These results demonstrate the importance of considering the interplay between utility, informativeness, and complexity in language evolution, and suggests a principled, cognitively motivated framework for emergent communication in artificial agents.

In this paper, we review some of the relevant background that we build upon in developing our theoretical framework and methods. Experiments in three domains indicate the strengths and generalizability of our approach and suggest important directions for future research.

## BACKGROUND

In this section, we provide an overview of the relevant technical background for our work. First, we review IB systems, including IB analysis measuring the tradeoff between complexity and informativeness in human naming systems, and variational methods for training neural nets with the IB objective. Second, we review the technical details of the Vector-Quantized Variational Autoencoder (VQ-VAE), a neural network architecture for learning discrete representations in a continuous space; our VQ-VIB method combines ideas from VQ-VAE with concepts from IB. Third, we summarize related literature in Emergent Communication, highlighting how prior literature often uses a utility-based framework for inducing communication.

### The Information Bottleneck for Semantic Systems

Our work extends Zaslavsky et al. (2018)’s information-bottleneck framework for semantic systems, which we review in this section. In this framework, a speaker and listener optimize the IB tradeoff between informativeness and complexity of communication.^1^

In IB semantic systems, we assume a probabilistic source over meanings that a speaker wishes to encode: *m* ∼ ℙ(*m*). A meaning, *m*, represents a belief state, or a probability distribution over possible inputs. A speaker is characterized as a probabilistic encoder mapping from *m* to communication *w: q*(*w*|*m*). Conversely, a listener seeks to recover a reconstructed meaning from communication: $\hat{m}=q\left(m|w\right)$. Both speakers and listeners are characterized as probabilistic, with deterministic versions a subset of the more general stochastic view.

Within this framework, one may measure the complexity and informativeness of communication. Complexity is measured as the mutual information between the speaker’s inputs and communication: *I*(*m*;*w*). Intuitively, this corresponds to the number of bits allocated for communication. Simultaneously, informativeness corresponds to notions of similarity between the speaker’s and listener’s belief states and can be measured via the negative expected Kullback-Leibler (KL) divergence: $-E\left[D_{KL}\left[m∥\hat{m}\right]\right]$. Lower KL divergence values arise from more similar distributions, so decreasing the KL divergence leads to a listener “understanding” a speaker better.

Zaslavsky et al. (2018) propose that, in natural language naming systems and in expectation over a speaker’s meanings, the speaker and listener jointly optimize the encoder and decoder functions, *q*(*w*|*m*) and *q*(*m*|*w*), according to a tradeoff between complexity and informativeness, modulated by a scalar parameter *β* > 0:

$$\mathrm{minimize}\;I_{S}\left(m;w\right)+\beta E_{S}\left[D_{KL}\left[m∥\hat{m}\right]\right]$$ (1)

This theoretical IB tradeoff, where *β* represents the importance afforded to informativeness relative to complexity, yields two important insights: 1) at least in simple systems, one can derive optimal communication schemes for varying *β*, and 2) one can compare human naming systems to IB-optimal systems by measuring their informativeness and complexity values (where IB-optimal systems achieve maximum informativeness for a given complexity level). In a variety of works, covering diverse semantic domains (e.g., colors, pronouns, containers, etc.) and hundreds of languages, human languages are consistently near-optimal in the IB sense, while exhibiting different *β* tradeoff values (Mollica et al., 2021; Zaslavsky et al., 2018, 2019, 2021). That is, each language achieves near-maximal informativeness for its complexity level, but different languages settle on different complexity levels.

While the IB tradeoff characterizes important aspects of communication, it fails to capture task-relevant information. In the driving scenario introduced earlier, optimizing for the informativeness of two-word communication systems would lead passengers and drivers to divide all driving angles into two even halves (Figure 1b and c). Such divisions could support somewhat generalizable communication (e.g., learning concepts of left vs. right) but ignore task-specific utility functions (in our example, maintaining the steering wheel within angle *θ*). Thus, IB tradeoffs produce task-agnostic communication systems.

### The Variational Information Bottleneck

While exact computation of IB tradeoffs is possible in simple systems, typical approaches fail to scale to complex settings; the Variational Information Bottleneck (VIB) is a scalable variational approximation method for IB. As introduced by Alemi et al. (2017), VIB comprises a stochastic neural network encoder and decoder. In VIB, a neural encoder with weights *θ, q_θ_*(*z*|*x*), maps input *x* to parameters of a *d*−dimensional Gaussian distribution—*μ*(*x*) and Σ(*x*)—from which a continuous latent variable, *z* ∈ ℝ*^d^*, is sampled. A neural decoder with weights *ϕ, q_ϕ_*(*y*|*z*), reconstructs a target feature, *y*, from *z*. In standard VIB literature, the encoder and decoder are jointly trained according to a tradeoff between decoder accuracy and the complexity of representations but, rather than using *I*(*x*;*z*) directly, VIB uses a variational bound on complexity:

$$I_{q_{\theta }}\left(x;z\right)\leq E\left[D_{KL}\left[q_{\theta }\left(z|x\right)∥r\left(z\right)\right]\right]$$ (2)

which holds for any distribution *r*(*z*) (typically set to $N\left(0,I_{d}\right)$). Overall, therefore, the VIB objective is:

$${\mathrm{maximize}}_{\phi ,\theta }\;I_{q_{\phi }}\left(z;y\right)-\beta I_{q_{\theta }}\left(x;z\right)$$ (3)

$$\leq I_{q_{\phi }}\left(z;y\right)-\beta E\left[D_{KL}\left[q_{\theta }\left(z|x\right)∥r\left(z\right)\right]\right]$$ (4)

This closely resembles Zaslavsky et al. (2018)’s IB framework for semantic systems, with tradeoffs between complexity and informativeness (although note some flipped signs and the role of *β*, due to terms for informativeness vs. distortion, and maximization vs. minimization). Unlike in standard IB, however, VIB uses variational bounds for complexity and, depending upon the predicted feature, *y*, for informativeness as well. The flexibility of VIB has in turn supported applications of Information Bottleneck methods across disciplines, including economics (Aridor et al., 2024) and modeling of human intelligence (Malloy, 2022).

### Vector-Quantized Variational Autoencoder

While VIB generates complexity-limited continuous representations, the Vector-Quantized Variational Autoencoder (VQ-VAE, van den Oord et al., 2017) generates discrete, but potentially highly complex, representations. VQ-VAE models comprise a neural network encoder and decoder, mediated by a *d*−dimensional latent space. A codebook of *K* vectors, *ζ_i_* ∈ ℝ*^d^, i* ∈ [1, …, *K*], defines a set of learnable discrete representations within the latent space. Notably, *K*, as set when initializing a VQ-VAE neural network, specifies a fixed codebook size. To generate a latent representation of an input, *x*, a deterministic encoder maps from *x* to a continuous latent representation *z*(*x*) ∈ ℝ*^d^*, which it then discretizes by selecting the index of the closest element of the codebook: *i* = argmin*_j_* ‖*z*(*x*) − *ζ_j_*‖^2^. The final discrete representation is this closest element, *ζ_i_*(*x*). Given *ζ_i_*(*x*), a deterministic decoder network seeks to reconstruct the encoder’s input.

During training, the weights of the encoder, the decoder, and the codebook are updated using gradient descent; we represent these weights as θ. Passing gradients through the non-differentiable discretization process (specifically, the argmin operation) is challenging; VQ-VAE uses a straight-through estimator, a common method for estimating gradients through non-differentiable processes. VQ-VAE is trained according to the loss function in Equation 5, combining the evidence lower bound (ELBO) with two vector-quantization terms that encourage continuous embeddings and codebook elements to cluster.

$$l_{VQ-VAE}=\log p\left(x|\zeta_{i}\left(x\right);\theta \right)+∥sg\left[z\left(x\right)\right]-\zeta_{i}\left(x\right){∥}^{2}+\alpha ∥z\left(x\right)-sg\left[\zeta_{i}\left(x\right)\right]{∥}^{2}\;$$ (5)

Here, the first term represents the evidence lower bound (ELBO), a measure of the estimated likelihood of training data. The second and third terms are clustering losses (using sg to stand for the stopgradient operator) that cause continuous embeddings and codebook elements to move closer together in the latent space. Lastly, *α* is a scalar tradeoff hyperparameter controlling the relative importance of clustering terms, typically set to 0.25 (van den Oord et al., 2017).

Overall, VQ-VAE models learn discrete representations in a continuous space (the codebook elements) that enable high-quality reconstructions of inputs. Our work builds upon aspects of the VQ-VAE architecture but differs both in training objective and implementation. By introducing complexity bounds in training and using a different neural architecture to support such bounds, our VQ-VIB methods allow us to vary the complexity of communication, which we show enables more human-like communication. In particular, because VQ-VAE models are deterministic, they do not support complexity training losses.

### Emergent Communication

Our work combines ideas of IB and discrete representation learning (reviewed in the previous two sections) with the utility-based framing of emergent communication. In traditional EC work, agents are trained in cooperative multi-agent environments to maximize a utility function (sometimes called the reward) (Havrylov & Titov, 2017; Kottur et al., 2017; Lazaridou et al., 2018; Lowe et al., 2017; Wang et al., 2020). In partially-observable environments, endowed with “cheap-talk” channels that allow agents to broadcast vectors to each other, agents often learn to communicate relevant information to each other. In our driving example, communication between a passenger and an inattentive driver will emerge by the passenger learning to broadcast information about acceptable steering wheel angles and the driver simultaneously learning to interpret such communication (Figure 1a). Critically, the utility pressure in EC is distinct from an informativeness pressure, which generates task-agnostic communication.

The utility-based EC framework is a powerful mechanism for inducing task-specific communication: in various settings, agents may coordinate in simulated environments to find target locations (Lowe et al., 2017), or avoid collisions in simulated road intersections (Chaabouni, Strub, et al., 2021). Despite the flexibility of such methods, traditional EC agents often exhibit undesirable properties, such as being overly-complex (Chaabouni et al., 2019), slow to converge (Eccles et al., 2019; Lin et al., 2021), or unable to generalize to novel inputs (Kottur et al., 2017). As an example of overly-complex communication, whereas humans might use only a small number of words to categorize colors, agents might output a distinct communication vector for each color.

Based in part by a desired to mirror the complexity-limited discrete nature of words, some recent EC works seek to induce complexity-limited discrete communication among agents (Kottur et al., 2017). In recent discrete EC works, agents communicate via onehot vectors, and the dimensionality of these vectors specifies the maximum vocabulary size (Chaabouni, Strub, et al., 2021; Lowe et al., 2017; Rita et al., 2020). In some works, therefore, authors decrease the vocabulary size to a small number *k*, which sets a maximum communication complexity limit of log_2_(*k*) bits. Beyond such hard-coded limits on complexity, two recent studies of discrete EC add corrupting noise to communication which affects communication complexity through environmental, rather than agent architecture, choices (Kuciński et al., 2021; Tucker et al., 2021).

Lastly, Lin et al. (2021) indirectly explores the role of informativeness in guiding emergent communication, by using a reconstruction loss to generate communication encodings. This reconstruction loss encourages communication to contain more decodable information and is closely aligned with notions of informativeness. The authors find that their method tends to induce faster convergence (i.e., the team performs well at a task faster), but they note that their method might induce unnecessarily complex communication.

## INFORMATION-CONSTRAINED EMERGENT COMMUNICATION

Our technical contributions are twofold: first, we introduce an information-constrained emergent communication (ICEC) framework that incorporates a utility-based loss into the IB informativeness-complexity tradeoff; second, we propose a neural network method, named the Vector Quantized Variational Information Bottleneck (VQ-VIB), which may be trained within our framework.

**Figure 2.**
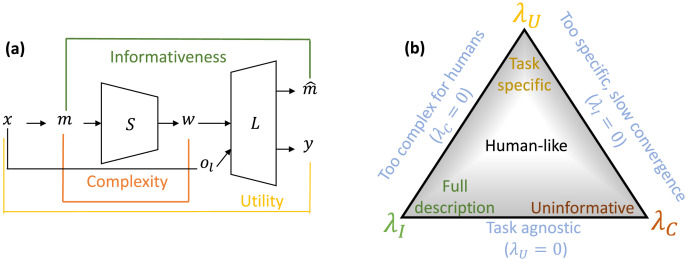
(a) A general communication model in which a speaker (*S*) and a listener (*L*) learn to communicate by trading off utility, informativeness, and complexity (see main text for further detail). (b) The three tradeoff parameters, (*λ_U_*, *λ_I_*, *λ_C_*), control a variety of important communicative behaviors that can be mapped on a simplex. The region in the middle of the simplex, rather than the extremes, is expected to capture human-like communication.

The ICEC framework extends traditional utility-based EC to include terms for informativeness and complexity. Consider a simple EC setting with two agents: a speaker and a listener (*S* and *L*), depicted in Figure 2a. Given a global state, *x*, the speaker receives a (potentially noisy) partial observation of the state and encodes it as a meaning, *m*, representing a belief state or probability distribution over *x*. The speaker stochastically maps *m* to an output communication vector, *w: w* ∼ *S*(*w*|*m*). Based on *w* and its own partial observation of the state (*o*_*l*_), the listener simultaneously reconstructs the meaning ($\hat{m}$) and takes an action *y* ∈ *Y: y* ∼ *L*(*y* |*w*, *o*_*l*_). The utility of actions is measured as a function of the state and the listener’s action, *U*(*x*, *y*).^2^

Figure 2a shows not only how a speaker and listener can coordinate to learn a communication protocol, but also how functions of different terms in the communication process reflect important quantities like informativeness and complexity. Just as IB optimization depends upon the tradeoff between informativeness and complexity, regulated by a scalar parameter *β*, we consider a maximization of three terms: utility, informativeness, and complexity. Thus, the ICEC objective is:

$$\mathrm{maximize}\;\lambda_{U}E\left[U\left(x,y\right)\right]-\lambda_{I}E\left[D_{KL}\left[m∥\hat{m}\right]\right]-\lambda_{C}I_{S}\left(m;w\right),$$ (6)

where *λ_U_* represents the scalar weight for increasing utility, *λ_I_* for increasing informativeness (or, equivalently, minimizing distortion), and *λ_C_* for minimizing complexity.

The relative weights of these three scalar terms dictate important properties of optimal emergent communication, as depicted in Figure 2b. For example, setting *λ_C_* too high might lead to uninformative communication, but setting *λ_C_* = 0 might lead to overly complex communication. Similarly, *λ_U_* controls the task specificity of communication, and *λ_I_* controls a task-agnostic measure of informative communication. In our experiments, we typically fixed some *λ* values while varying others, but one could also adopt a simplex formulation wherein the sum of all *λ* remain fixed.

Beyond the individual pressures reflected by each term in the ICEC framework, the tensions between terms reveal important tradeoffs likely present in human communication. First, tradeoffs between informativeness and complexity dictate important aspects of task-agnostic compression; such compression has been widely used in studying human naming systems (Mollica et al., 2021; Zaslavsky et al., 2018, 2019, 2021). Second, maximizing utility while minimizing complexity corresponds to the canonical Information Bottleneck (IB) optimization of retaining task-relevant information in a lossy compression framework (Tishby et al., 1999). Notably, this is distinct from informativeness-complexity tradeoffs insofar as a utility function corresponds to a task-specific metric. In the simplified driving example in the Introduction, for example, a utility pressure would differentiate between acceptable angles, *θ*, that an informativeness pressure, based only on the ability to reconstruct any angle, could discard. Other works explore how complexity bounds limit expected utility in Markov Decision Processes, where agents accrue reward based on states and actions but penalties based on highly deterministic and specific (complex) policies (Rubin et al., 2012; Tishby & Polani, 2011).

Lastly, informativeness and utility, while not in direct competition, represent distinct theories of language development. Does language emerge to accomplish some task (utility) or to convey general meanings (informativeness)? In the absence of complexity limits, one may simultaneously optimize utility and informativeness through highly complex communication that includes all information about a speaker’s belief. In complexity-limited communication, however, the relative weights of *λ_U_* and *λ_I_* dictate tradeoffs of task-specific and task-agnostic communication. Overall, we believe that human communication is likely driven by each of the three terms we consider and that, to induce the most human-like emergent communication, one should therefore explicitly model the tradeoffs between these terms.

### Vector-Quantized Variational Information Bottleneck

Equation 6 trades off terms for utility, informativeness, and complexity, but directly solving this maximization is intractable in large domains (Alemi et al., 2017). Therefore, we train neural network agents to maximize a tractable variational bound of the same objective.

Our method, named the Vector-Quantized Variational Information Bottleneck, or VQ-VIB, is a variational method for learning complexity-limited discrete representations in a continuous space. Intuitively, VQ-VIB combine notions from the variational information bottleneck to limit complexity with vector quantization for discretization. While VQ-VIB borrows from prior art, it is distinct in several important ways. Unlike VQ-VAE, it uses a stochastic encoder, which is necessary for variational bounds on complexity, and unlike VIB, it generates discrete representations.

**Figure 3.**
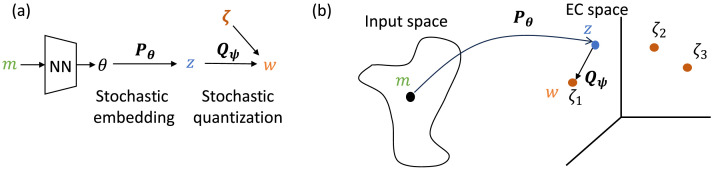
a) VQ-VIB employs a stochastic embedder (*P_θ_*) that samples a continuous embedding, *z*, based on parameters extracted from a meaning, *m*, and a stochastic quantizer (*Q_ψ_*) that maps *z* to a discrete word, *w*, using a codebook, *ζ*. The neural net mapping from *m* to *θ*, and the codebook *ζ* are learnable parameters. b) The two-stage process first maps from a potentially high-dimensional input space into an embedding space, where discrete vectors, *ζ*, are used for quantization. In this work, we propose two implementations of VQ-VIB by providing different *P_θ_* and *Q_ψ_*.

Our general VQ-VIB method combines a stochastic embedding mechanism with stochastic discretization, as depicted in Figure 3a. First, given a meaning, *m*, we generate parameters of a probability distribution, *θ*, from which a continuous latent embedding, *z* is sampled: *z* ∼ *P_θ_*(*m*). Next, *z* is quantized (i.e., set to a discrete vector) via a stochastic process to generate a discrete embedding, *w* ∼ *Q_ψ_*(*z*). The neural network that produces *θ*, as well as the set of discrete embeddings, *ζ*, are parametrized by learnable weights. Note that the VQ-VIB method is a strict generalization of VQ-VAE; deterministic embedding or quantization functions are a special case of stochastic functions, and having at least one stochastic function supports variational bounds on complexity.

The changes in representation formats in VQ-VIB are depicted graphically in Figure 3b. First *P_θ_* maps from a high-dimensional input space into the EC space. Within the EC space, we assume VQ-VIB models have *K* learnable codebook elements: *ζ_i_, i* ∈ [1, *K*]; *ζ* = [*ζ*_1_, *ζ*_2_, …, *ζ_K_*]. (In the diagram, *K* = 3.) Lastly *Q_ψ_* stochastically maps from the continuous representation, *z*, to one of *ζ_i_*, to produce the final output *w*. In this paper, we propose two different implementations of VQ-VIB, which differ by providing different implementations of *P_θ_* or *Q_ψ_*.

#### Vector-Quantized Variational Information Bottleneck – Normal Distribution.

Our first encoder architecture, named the Vector-Quantized Variational Information Bottleneck – Normal, or VQ-VIB_𝒩_, draws its name from the normal distribution it uses for *P_θ_*. That is, $z∼N\left(\mu \left(m\right),\Sigma \left(m\right)\right)\in {\mathbb{R}}^{d}$. This stochastic encoding process via a normal distribution is similar to standard VIB parametrizations (Alemi et al., 2017). Next, VQ-VIB_𝒩_ deterministically discretizes *z* by selecting the closest element of the codebook, *ζ*. That is, *Q_ψ_* is set to the argmin operation over distance in the EC space. This quantization step is borrowed from VQ-VAE literature. Overall, the VQ-VIB_𝒩_ encoder is a probabilistic function given by:

$$S_{N}\left(w|m\right)=\mathbb{P}\left(w=\underset{\zeta_{i}\in \zeta }{\text{argmin}}{\left\|z\left(m\right)-\zeta_{i}\right\|}^{2}\right)$$ (7)

where *w* is a discrete communication vector, *m* is the speaker’s meaning, *z*(*m*) is the continuous latent variable sampled from the Gaussian distribution, and *ζ* is the codebook.

#### Vector-Quantized Variational Information Bottleneck – Categorical Distribution.

Our second encoder architecture, named the Vector-Quantized Variational Information Bottleneck – Categorical, or VQ-VIB_𝒞_, differs from VQ-VIB_𝒩_ by its implementations of *P_θ_* and *Q_ψ_*. First, *P_θ_* is a deterministic function based on a feedforward encoder, rather than sampling from a Gaussian: *z*(*m*) = *P_θ_*(*m*). VQ-VIB_𝒞_ uses a stochastic *Q_ψ_*, however, by sampling a discrete representation according to probabilities based on the negative Euclidean distance from *z* to each *ζ_i_*. That is,

$$S_{C}\left(w=\zeta_{i}|m\right)\propto e^{-{\left\|z\left(m\right)-\zeta_{i}\right\|}^{2}},$$ (8)

which generates a probability distribution by normalizing for all *ζ_i_* ∈ *ζ*.

Overall, VQ-VIB_𝒩_ and VQ-VIB_𝒞_ reflect two different interpretations and implementations of the same broad idea. Both fit within the general VQ-VIB method by providing implementations of *P_θ_* and *Q_ψ_*. The difference in sampling mechanisms can be interpreted as uncertainty over a continuous semantic space (for VQ-VIB_𝒩_) that is deterministically discretized, or uncertainty over how to discretize a deterministically-generated continuous embedding (for VQ-VIB_𝒞_). Future work may propose alternate VQ-VIB implementations via different sampling or embedding methods within our framework.

Regardless of the architecture, while VQ-VIB models are instantiated with *K* learnable codebook elements, one may vary the *effective* codebook size of a given model via complexity bounds. As demonstrated in experiments, penalizing the complexity of communication leads to agents using fewer distinct tokens. We view this soft pressure on learning smaller codebooks as more cognitively plausible than hardcoding lower *K* values.

Lastly, we note that both VQ-VIB_𝒩_ and VQ-VIB_𝒞_ support a combinatorial token architecture that increases the codebook size of models without increasing the number of parameters in the network. Intuitively, rather than generating a single continuous representation in ℝ*^d^*, an encoder can generate *n* representations in ${\mathbb{R}}^{\frac{d}{n}}$ and discretize each of those representations. Full discussion of this method is included in Appendix A; conceptually this change in architecture does not alter the underlying variational bounds or discrete nature of encodings. Supporting a greater number of representations enables more complex and informative communication but, as we show in experiments, leads to less “human-like” communication.

#### Learning Objective.

Regardless of the specific architecture, VQ-VIB agents can be trained according to variational bounds of the ICEC objective defined in Equation 6.

We used the same bound on informativeness (the second term in Equation 6) for both VQ-VIB architectures. Assuming that true states, *x* ∈ ℝ*^n^*, are corrupted by zero-mean Gaussian noise with some variance Σ, belief states, *m*, are given by $m=N\left(x,\Sigma \right)$. Under this assumption, $E\left[D_{KL}\left[m∥\hat{m}\right]\right]\leq \frac{1}{2}E\left[{\left\|m-\hat{m}\right\|}^{2}\right]+\text{const}$. Thus, training a simple decoder, *D*, that outputs a reconstructed meaning based on communication, $\hat{m}=D\left(w\right)$, provides an upper bound on informativeness.

For complexity (the third term in Equation 6), we used architecture-specific variational bounds. For VQ-VIB_𝒩_,

$$I_{S}\left(m;w\right)\leq I_{S}\left(m;z\right)\leq E\left[D_{KL}\left[q_{\theta }\left(z|m\right)∥r\left(z\right)\right]\right]]$$ (9)

The first inequality follows from the data-processing inequality, and the second follows standard VIB bounds for any marginal distribution, *r*(*z*). In our implementations, we set $r\left(z\right)=N\left(0,I\right)$. Thus, for VQ-VIB_𝒩_, we bottleneck the sampling process prior to discretization, which in turn limits the complexity of the downstream, discrete representation.

For VQ-VIB_𝒞_, complexity is bounded via

$$I_{S}\left(m;w\right)\leq E\left[D_{KL}\left[q_{\theta }\left(w|m\right)∥r\left(w\right)\right]\right]$$ (10)

This bound differs from the one for VQ-VIB_𝒩_ by measuring the stochasticity the discretization process via *Q_ψ_*, whereas VQ-VIB_𝒩_ used *P_θ_*. Given the categorical distribution for *q_θ_*(*w*|*m*), *r*(*w*) represented a categorical prior over codebook elements, set in our experiments to a uniform distribution.

Combining terms for informativeness and complexity, the overall variational bound of the ICEC optimization in Equation 6 is

$$\mathrm{maximize}\;L_{\mathrm{var}}=\lambda_{U}E\left[U\left(x,y\right)\right]-\lambda_{I}E\left[{\left\|m-\hat{m}\right\|}^{2}\right]-\lambda_{C}I_{\mathrm{var}}\left(m;w\right),$$ (11)

where *I*_var_ is the architecture-specific variational bound on complexity, defined in Equation 9 for VQ-VIB_𝒩_ and Equation 10 for VQ-VIB_𝒞_.

Lastly, we trained VQ-VIB models by combining the ICEC variational bound, $L_{\mathrm{var}}$ with prototype clustering losses from VQ-VAE methods (as introduced in Equation 5) and a tie-breaking entropy loss:

$$\mathrm{maximize}\;L_{\mathrm{var}}-∥sg\left[z\left(m\right)\right]-\zeta_{i}\left(m\right){∥}^{2}-\alpha ∥z\left(m\right)-sg\left[\zeta_{i}\left(m\right)\right]{∥}^{2}-{\varepsilon \lambda }_{C}ℍ\left(w\right)$$ (12)

The final entropy term, ℍ(*w*) represents the estimated entropy over the codebook; penalizing high-entropy communication (weighted by a small scalar value, *ε*, times *λ_C_*) biased agents towards more human-like naming systems for a given complexity class. Further discussion of the entropy term is included in Appendix B.

## EXPERIMENT DESIGN

In a series of experiments, presented in the subsequent sections, we sought to characterize important aspects of information-constrained emergent communication and our VQ-VIB method. First, could VQ-VIB agents learn IB-optimal communication, as in human naming systems in simple domains? Second, in open-domain communication, with an unbounded set of possible objects to refer to, what are the roles of informativeness and complexity pressures on generalization (the ability to refer to novel objects) and similarity to human languages? Third, beyond object-naming experiments, could artificial agents learn similar IB tradeoffs in simulated grounded settings?

**Figure 4.**
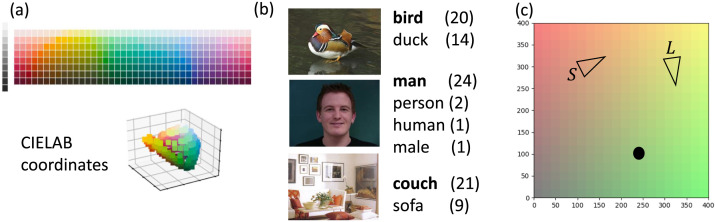
We conducted experiments in 3 domains: (a) a color-naming reference game, (b) a reference game of natural images, accompanied by human-provided names, and (c) a 2D navigation environment.

We studied these questions in three experimental domains, depicted in Figure 4. Overall, we found that VQ-VIB agents, trained within the ICEC framework, learned similar complexity-informativeness tradeoffs to humans. In all domains, encouraging informativeness led to faster convergence (agents learning meaningful communication), but penalizing complexity was important for inducing more human-like communication. Lastly, we observed consistent trends across all three domains, indicating the generality and robustness of our ICEC training framework.

## EXPERIMENT 1: EMERGENT COLOR NAMING SYSTEMS

In our first experiment, we considered whether VQ-VIB agents could learn IB-optimal communication systems in a color reference game, depicted in Figure 4a, and whether doing so would induce aspects of human-like communication. Color-naming has been central to many cognitive theories of semantics, as languages must encode the continuous color spectrum into a finite set of words (Berlin & Kay, 1969; Regier et al., 2007; Steels & Belpaeme, 2005). Data in the World Color Survey (WCS) demonstrate how 110 nonindustrialized societies name color, providing a large corpus of human data (Kay et al., 2009). Thus, we tested our ICEC framework for training agents to communicate about color, and compared the resulting EC agents to human naming systems and analytically-computed IB systems.

### Experiment Setup

**Figure 5.**
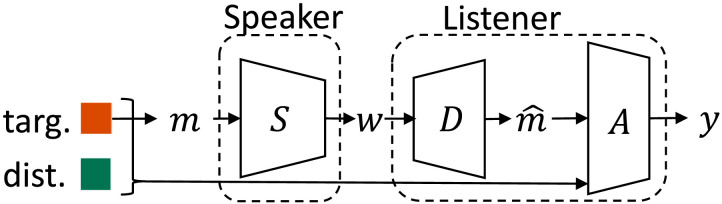
Reference game setup for our color experiment. Given a noisily-observed target color, the speaker communicated to a listener, which predicted, from a set of the target and a distractor color, the target. Utility was based on team accuracy.

We trained a team of agents, comprising a speaker and a listener, in a reference game, as depicted in Figure 5 (Lewis, 2008). A speaker observed a randomly-drawn target color (in our figure, a reddish-orange color), corrupted by Gaussian noise, and emitted communication, *w*. (The CIELAB representation of colors, the prior over which colors were sampled, and the observation noise were borrowed from Zaslavsky et al. (2018) and more broadly inspired by prior work in human color perception (Chaabouni, Kharitonov, et al., 2021; Mokrzycki & Tatol, 2011). That communication vector was passed to a listener agent, comprising a decoder, *D*, that reconstructed the speaker’s observation, and an actor, *A*, that observed the reconstruction as well as the target and a distractor color. The listener had to predict which of the two candidates was the target color.

Achieving high team accuracy (correctly identifying the target color) given a complexity-limited communication channel requires the speaker and listener to develop a shared understanding of communication about color.

Throughout our experiments, we set *λ_U_* = 1.0, *λ_I_* = 1.0; this tended to lead to highly accurate team performance for low *λ_C_*. After team convergence (40 epochs), we incrementally increased *λ_C_* by a fixed step size every training episode for 50 epochs, which induced a spectrum of communication complexity levels.

### Results

**Figure 6.**
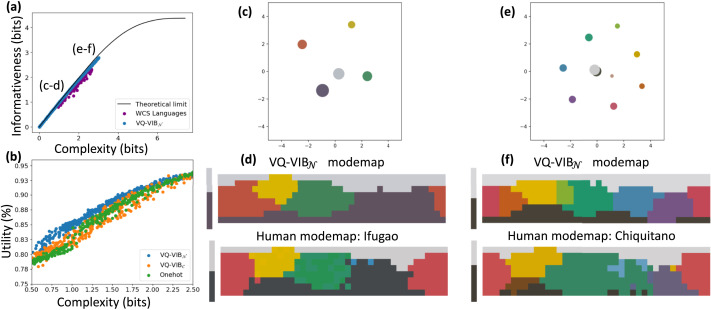
Color reference game results. Within the ICEC framework, we annealed *λ_C_* to generate a spectrum of near-IB-optimal naming systems, closely matching human-like diversity (a). Within the range of human-like complexities, VQ-VIB_𝒩_ tended to achieve greater utility, for the same complexity, as other methods (b). At low (c–d) and high (e–f) complexity levels, VQ-VIB_𝒩_ used fewer or more distinct tokens for colors, and closely aligned with human languages at similar complexity levels. Notably, the tokens were embedded in a semantically-meaningful communication space, as visualized using 2D PCA (c, e).

We found that controlling the complexity of communication enabled EC agents to learn human-like color naming systems, and that VQ-VIB agents learned semantically-meaningful communication spaces. Visualizations of our results, for VQ-VIB_𝒩_, are included in Figure 6.

Figure 6a shows how VQ-VIB_𝒩_ EC messages were nearly optimally-efficient, just under the IB-optimal curve for informativeness vs. complexity. Furthermore, by varying *λ_C_* during training, VQ-VIB_𝒩_ communication spanned the range of complexities observed in human color naming systems recorded in the WCS dataset. Crucially, this shows that VQ-VIB agents can learn IB-optimal communication and can be controlled similarly to analytical IB methods.

Snapshots of VQ-VIB_𝒩_ naming systems, at different complexity levels, are shown in Figure 6c–f. At low complexity (c and d), agents used only 5 communication vectors, representing high-level color categories. At high complexity (e and f), agents used more communication vectors and partitioned the color space more finely. By changing the complexity of EC, VQ-VIB_𝒩_ agents learned communication systems similar to different human naming systems (e.g., Ifugao at low complexity and Chiquitano at high complexity).^3^ Videos of communication evolution, for VQ-VIB_𝒩_ and VQ-VIB_𝒞_ are available online, showing similar behavior for both model types, and smooth interpolation of behavior across complexity levels.

Beyond matching human languages, VQ-VIB_𝒩_ learned a meaningful communication space. 2-dimensional principal component analysis (PCA) of the communication space at different complexity levels (Figure 6c and e) show how vectors representing similar colors were located in similar locations in the communication space. For example, the embedding for orange-like colors is close to the embedding for yellow colors, and far from the embedding for blue colors. Thus, we claim that, at least intuitively, VQ-VIB models have learned a semantically-meaningful embedding space: similar inputs (per human judgements) lead to similar communication vectors. This meaningful embedding space likely supported VQ-VIB_𝒩_’s improved utility relative to other methods (Figure 6b, plotted for five training runs of each speaker architecture).

**Figure D.2:**
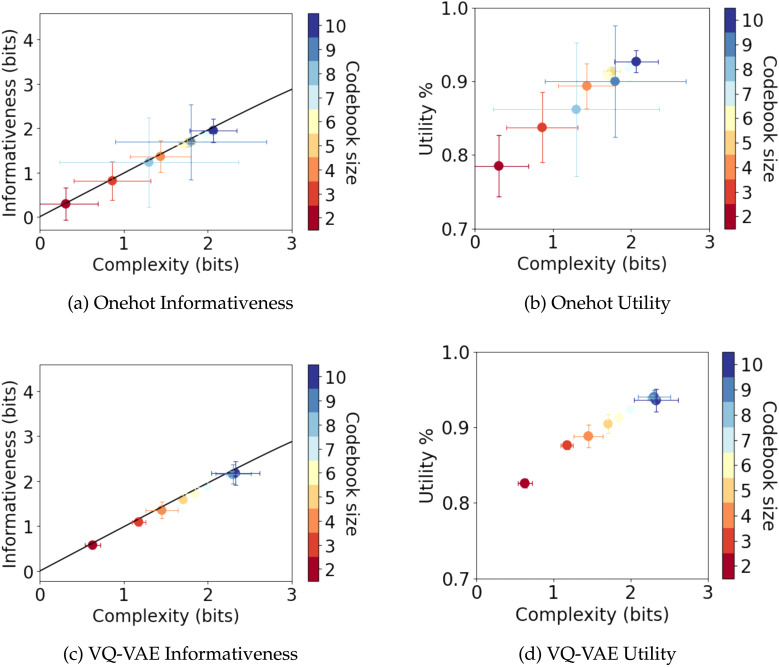
Informativeness and utility vs. complexity for onehot and VQ-VAE models, for different hardcoded codebook sizes. Without informativeness or complexity pressures, varying the architecture was the only way to indirectly control important model behaviors.

In addition to comparing different variational methods within the ICEC framework, we trained baseline non-variational onehot and VQ-VAE models without informativeness or complexity losses. Without variational methods and information-theoretic pressures, we were only able to induce variations among agents by explicitly encoding different codebook sizes. Figure D.2 in Appendix D shows plots of resulting behavior.

Quantitative evaluation over five runs of these non-variational models showed that they were both less optimal (in the IB sense) and less “human-like” than our variational approaches. Table 1 includes results for comparing EC communication to the WCS languages (measured via the generalized Normalized Information Distance, or gNID) and deviation from the IB theoretical bound; lower values are better for both metrics. VQ-VIB_𝒩_ was both more human-like and more efficient than utility-based Onehot or VQ-VAE agents. Furthermore VQ-VIB_𝒩_ agents were more human-like (lower gNID) than standard IB methods which ignore utility measures (Zaslavsky et al., 2018). For completeness, we include these metrics for VQ-VIB_𝒞_ and a variational extension of traditional onehot communication in Table D.1. These methods achieve similar results to VQ-VIB_𝒩_ and confirm that variational methods, trained in the ICEC framework, are more efficient and human-like than traditional EC methods.

**Table 1.** Quantitative evaluation of emergent color naming systems with respect to the WCS languages (Kay et al., 2009), and the IB-optimal systems (Zaslavsky et al., 2018). Human-agent gNID measures the dissimilarity, on average, between the WCS systems and the nearest artificial systems; efficiency loss measures the average deviation from the IB theoretical limit of efficiency. For both measures, lower values are better. VQ-VIB_𝒩_ approximates the IB bound (low efficiency loss) well while yielding systems that are most human-like (low gNID), outperforming even the optimal IB systems.

	VQ-VIB_𝒩_	Onehot	VQ-VAE	IB-optimal
Human-agent gNID	**0.151 (0.00)**	0.42 (0.16)	0.38 (0.15)	0.18 (0.10)
Efficiency loss	**0.024 (0.00)**	0.06 (0.01)	0.08 (0.01)	NA, 0 by definition

Overall, results from this domain indicate that agents, trained in our ICEC framework, learn to communicate according to similar IB tradeoffs found in human naming systems. Furthermore, VQ-VIB models in particular learn meaningful embedding spaces that represent semantic relationships.

## EXPERIMENT 2: OPEN-DOMAIN COMMUNICATION

Beyond studying communication a color-naming domain, we considered how the ICEC framework influenced learned communication in a richer domain. Specifically, we used the ManyNames dataset to study aspects of generalization and alignment with natural language embeddings (Silberer et al., 2020). In training agents, we found that encouraging informativeness led to higher self-play rewards (evaluating teams of agents that have trained together), including in harder evaluation settings in which agents communicated about types of images never seen during training. At the same time, limiting the complexity of communication was important for optimal human-agent alignment.

### Experiment Setup

We trained agents in a reference game using the ManyNames dataset (Silberer et al., 2020), which is particularly appropriate for studying alignment of EC and natural languages. It is composed of 25,000 images, each of which is annotated with roughly 36 English responses (we refer to Silberer et al. (2020) for details of data curation). Unlike most labeled image datasets with a closed set of prescribed labels, therefore, ManyNames reflects open-domain communication and captures important aspects of the probabilistic nature of human naming (Gualdoni et al., 2022; Mädebach et al., 2022). Examples of images in the dataset, with associated responses, are included in Figure 4b.

The images in Figure 4b reflect important characteristics of the dataset. First, there is a wide variety of the types of images; second, there is important variation in the naming data (Gualdoni et al., 2022; Mädebach et al., 2022). For example, while most participants labeled the top image in Figure 4b as a “bird,” others used the label “duck.” Both labels are correct but reflect different complexity levels. We hoped that, in training EC agents on this dataset and controlling the complexity of communication, we could induce human-like EC.

Beyond inducing similarities between EC and natural language, we were interested in how well EC generalized to novel inputs. Thus, rather than training on the full ManyNames dataset, we constructed semantically-distinct training- and test-sets from the full dataset. Before training, we recorded the most common response for each image (named the topname, and shown in bold in Figure 4). There were 442 topnames in the full dataset; we selected a random 20% of those names for the training set. All images with the matching topname were selected for the training set, while the test set was generated by finding all images for which *no* response matched a training-set topname. This train-test split procedure tended to produce semantically-distinct sets of similar sizes.

**Figure 7.**
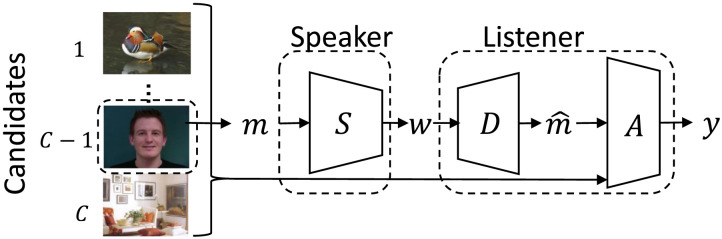
Reference game setup for ManyNames domain. The speaker observed a randomly-drawn candidate image and communicated to a listener, which decoded the speaker’s meaning and predicted which candidate image was the target.

We trained agents in the reference game setup shown in Figure 7. As in the color reference game, a speaker agent observed a noisy version of a target image drawn uniformly at random from the ManyNames dataset, and a listener agent had to identify the target among a set of *C* candidate images. During training, we set *C* = 2, but in some evaluation settings, we increased *C* to increase the difficulty of the task. Because of the high-dimensionality of the images, we used a pre-trained ResNet feature extractor to generate 512-dimensional representations for each images (He et al., 2016). These 512-dimensional vectors were passed through a pre-trained Variational Autoencoder (VAE) to simulate perceptual noise. Lastly, we note that during training candidate images were selected, by design, to always have distinct topname labels; this introduced an important distinction between utility (which could be maximized via unique words for each possible topname) and informativeness (which would be maximized at a much higher complexity level, representing fine-grained details in the target image).

During training, agents were trained via the ICEC losses, setting *λ_U_* = 1 and *λ_C_* = 0.01, with different *λ_I_* across trials to investigate the effect of informativeness pressures on communication. We found that these *λ* values tended to cover a range of interesting behaviors from at-chance accuracy (reflecting uninformative communication) to accuracy and complexity surpassing estimates based on English naming data. For a given set of *λ* values, we trained five teams from scratch with different random seeds, with each team training until convergence.

In evaluation, we studied both agent generalizability and aspects of EC-English alignment. To test generalizability, we measured team accuracy when evaluated on the held-out test set. Recall that these out-of-distribution (OOD) inputs were semantically distinct from the training images used by the team during training; by testing on this set, we captured one aspect of how generalizable the EC was to novel inputs.

Beyond generalization, we sought to measure alignment between pre-trained EC agents and GloVe embeddings (Pennington et al., 2014). Notions of “alignment” typically correspond to representational similarity of some sort: two aligned representation spaces might encode the same inputs in similar locations, for example (Moschella et al., 2023; Sucholutsky & Griffiths, 2023). In our work, we use two specific measures of alignment: functional alignment and relative representation alignment.

Our first metric, functional alignment, captured how well EC and GloVe embedding spaces could be aligned to perform well on a reference game. Intuitively, this corresponded to ideas of translation between the two spaces. Using a pre-trained EC speaker, we fit a linear mapping from EC vectors for images to GloVe embeddings of an English label for the image (drawn from the responses associated with each image in the dataset). We then evaluated team accuracy on the ManyNames reference game using a simulated English speaker (again, drawing responses associated with each image) and the pre-trained EC listener, mediated by the linear mapping. High team accuracy indicated that a linear mapping could capture important similarities between EC and GloVe embedding spaces.

Our second metric, relative representation alignment, reflected similarities in distances between encodings in different latent spaces (Moschella et al., 2023). Concretely, we sampled 100 random images (and for each image, a sampled English response, as before) to generate EC and GloVe embeddings. We then measured the pairwise distance between each embedding in each space; this generated 10000 distances in each space (counting duplicates of distances from A to B and B to A, for example). Lastly, we computed the Spearman correlation coefficient between these distances across the spaces. A large positive value would indicate that points that were close together in one space were close together in another space. Conversely, completely “unaligned” spaces should have correlation coefficients of 0. To address the additional stochasticity introduced by randomly sampling images for calculating alignment, we conducted ten evaluation runs and report the mean correlation coefficient.

Both alignment metrics indirectly measured the semanticity of VQ-VIB communication spaces. If an EC space achieves high alignment with the GloVe embedding space (which we state is a semantic space), then the EC space necessarily encodes semantically meaningful information. Thus, while rigorously defining how “meaningful” or “semantic” an EC space is remains challenging, notions of alignment with word embedding spaces offer important insights.

### Generalization Results

**Figure 8.**
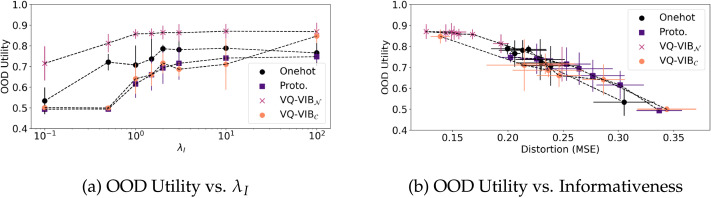
Team accuracy on OOD inputs as a function of *λ_I_* (a) or informativeness (b). By increasing *λ_I_*, we increased the communicative informativeness for all models, which in turn increased utility on OOD inputs.

In our generalization experiments, increased informativeness led to greater team accuracy on OOD inputs. Figure 8 shows team accuracy as a function of *λ_I_* and informativeness.

Jointly, the plots indicate how informativeness pressure induced greater OOD utility. Figure 7a shows that by increasing *λ_I_*, we increased OOD scores. Given different inductive biases, some model architectures increased their OOD scores more quickly as a function of *λ_I_* (e.g., VQ-VIB_𝒩_) and some architectures’ utility values plateaued as *λ_I_* grew larger.^4^ However, in general, all architectures tended to achieve greater utility with greater *λ_I_*. Recall that agents were evaluated on OOD inputs; increased scores from greater *λ_I_* indicate an important generalization benefit of informativeness pressures.

Figure 8b reveals an even closer relationship between informativeness and OOD utility. Each point represents the mean distortion (inversely related to informativeness) and utility for a particular model architecture and *λ_I_*. By increasing *λ_I_*, distortion tended to decrease (left along the *x* axis) and utility increased (up along the *y* axis). The close relationship between informativeness and utility across architectures also explains model performance differences: while all models tended to achieve similar utility for the same informativeness, some models were not able to learn highly-informative communication, which limited performance.

**Figure 9.**
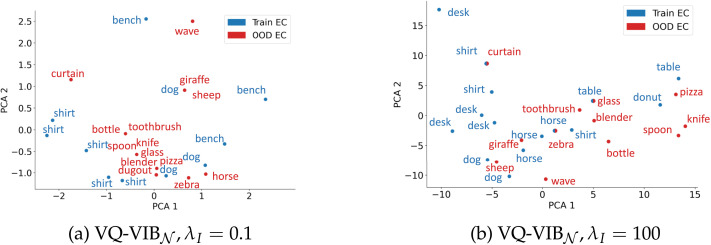
Visualization of VQ-VIB_𝒩_ communication spaces at low (a) or high (b) informativeness. Each point represents the 2D PCA projection of the EC token for training inputs (blue) or OOD inputs (red). At low informativeness, there were few commonly-used tokens, and the meanings associated with each token was highly stochastic. At higher informativeness, distinct images were more often encoded differently, and the communication space was semantically-interpretable, which often generalized to OOD inputs.

Lastly, visualization of VQ-VIB_𝒩_ communication space in Figure 9 indicates how agents generalized well to OOD inputs, and why increasing informativeness increased utility. For each token that an EC speaker emitted more than 1% of the time, we recorded which images caused the speaker to generate that token, and labeled the token with the most common topname among that set of images. We then repeated this process, using the same PCA projection, for OOD inputs, which we plotted in red.

These visualizations reveal two important characteristics of VQ-VIB_𝒩_ tokens. First, increasing the informativeness of communication increased the number of tokens used and the specificity of their meanings. For example, for *λ_I_* = 0.1, many of the tokens were most associated with images of shirts – this indicates a relatively stochastic sampling process that emitted different tokens for the same input. At high informativeness, however, tokens for more specific types of images (e.g., “donut” or “horse”) emerged. Second, the tokens seemingly formed a semantically-meaningful space that extended to OOD inputs. For example, in Figure 9b, the OOD input of an image of a sheep was located near training-set images of dogs and horses. Similarly, food-related images clustered near images of tables and donuts. This semantic embedding space, learned by both the speaker and the listener, supports generalization to novel inputs, similarly to how word embeddings improve natural language processing generalization to new words. This process also parallels child “overextension” phenomena, where young children apply known words to novel objects in a semantically-consistent way (Ferreira Pinto & Xu, 2021). (Although Figure 9 only displays a single VQ-VIB model’s embedding space, the next section, wherein we measure EC-NL alignment, corroborates the intuitions of similar images mapping to nearby EC tokens.)

For the results plotted in Figure 8, we evaluated OOD generalization with 2 candidate images at test time. As motivated by Chaabouni, Strub, et al. (2021), who advocate for evaluation in more challenging settings with more candidate images, we repeated such evaluations for *C* = 16 and *C* = 32. Generalization results in such settings are included in Appendix E. In general, we found that increasing *C* worsened team performance, as expected, and that VQ-VIB agents continued to outperform other architectures.

**Figure E.1:**
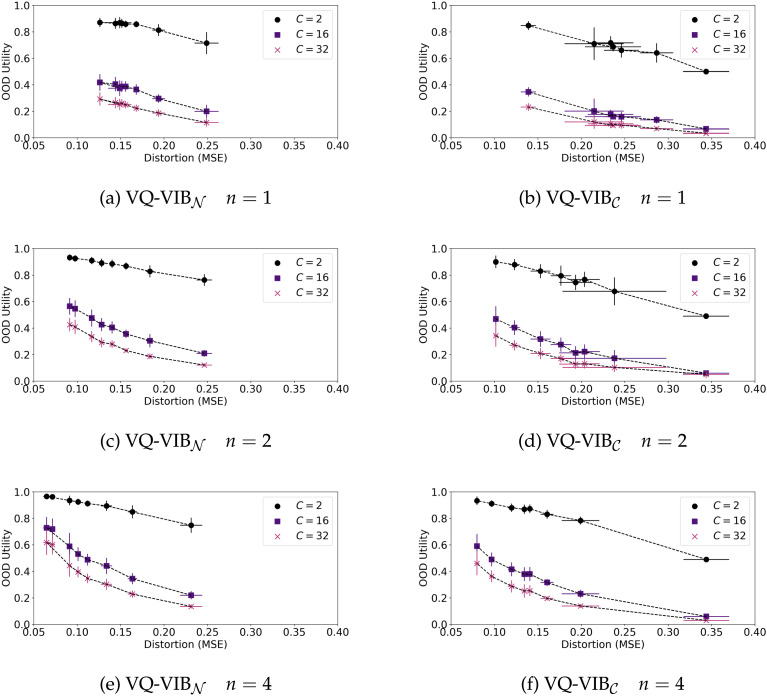
OOD accuracy vs. distortion for VQ-VIB_𝒩_ and VQ-VIB_𝒞_ (different columns) for different combinatorial codebook factors (different rows). For all architectures, OOD accuracy as distortion decreased, and using greater *n* supported lower distortion. Note how increasing *n* shifted all curves to the left, corresponding to lower MSE.

Lastly, we note that similar generalization trends hold, and are more obvious, for VQ-VIB agents with combinatorial codebooks. Recall that VQ-VIB_𝒩_ and VQ-VIB_𝒞_ support multiple discretizations that are concatenated together for a final communication vector. Increasing the number of concatenated vectors (*n*) decreased communication distortion, which improved OOD utility (see Appendix E, Figure E.1). For example, VQ-VIB_𝒩_ for *n* = 4 achieved OOD accuracy for *C* = 32 of roughly 60%, more than three times better than onehot or prototype agents in similar evaluation. Thus, our combinatorial codebook results corroborate trends from the main paper and show how combining tokens allows agents to develop more complex communication.

### Alignment Results

Using the same pre-trained agents from the prior experiments, we evaluated the functional alignment and relative representation alignment of EC agents with GloVe embeddings. In our functional alignment experiments, we fit a linear transform from EC tokens to GloVe embeddings and then evaluated the team accuracy for a simulated English speaker and an EC listener, mediated by this linear “translator.” In our relative representation alignment experiments, we computed the Spearman correlation coefficient between EC embedding distances and GloVe embedding distances.

**Figure 10.**
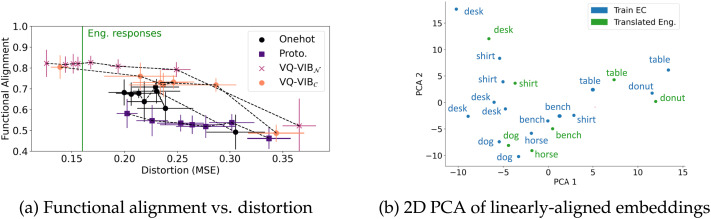
Functional alignment (a) and visualization of embeddings (b) for GloVe and EC models. Functional alignment improved as distortion decreased until reaching the estimated English informativeness, at which point performance plateaued.

Functional alignment, for different models as a function of informativeness, is plotted in Figure 10a. At high distortion (low informativeness), functional alignment improves as distortion decreases. This closely matches OOD trends. However, unlike in the generalization experiments, performance largely plateaued once EC informativeness decreased below English response informativeness. This indicates that performance was bottlenecked by the English speaker, and further informativeness simply added unnecessary complexity to the EC communication.

Visualization of translated GloVe embeddings in Figure 10b show similarities between the EC and GloVe embedding spaces. As before, each blue point represents an EC embedding for images from the training set. The green points show the embeddings generated by passing a GloVe embedding through the linear transformation. The semantic structure of the GloVe embeddings was largely preserved through this linear mapping, indicating substantial similarities between the EC embedding space and the semantically-meaningful GloVe space.

**Figure 11.**
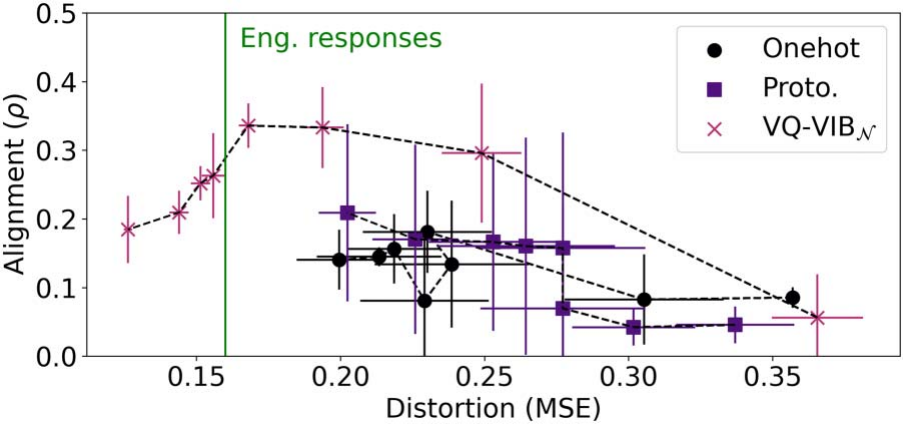
Relative representation alignment (*ρ*) between EC and GloVe embedding spaces. VQ-VIB_𝒩_ models had greater alignment than other models, and alignment peaked when EC model informativeness matched English response informativeness.

**Figure E.4:**
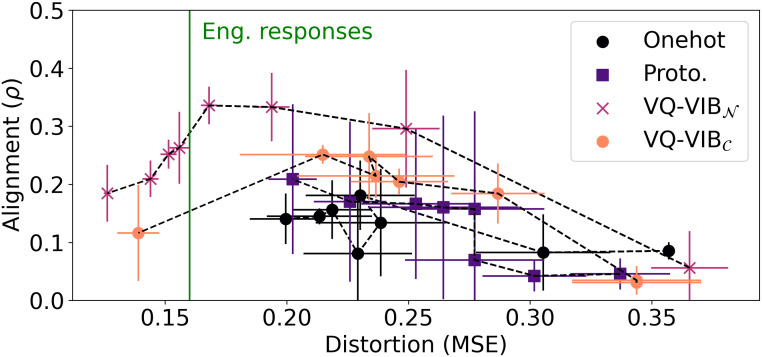
Relative representation alignment (*ρ*) between EC and GloVe embedding spaces. Results for VQ-VIB_𝒞_ models are overlayed over data from Figure 11. VQ-VIB_𝒞_ exhibits similar, although slightly worse, alignment trends to VQ-VIB_𝒩_.

Lastly, we measure relative representation alignment as a function of distortion and plotted results in Figure 11. This alignment metric demonstrates the importance of tuning EC informativeness to the right level even more starkly than the functional alignment experiments. At high distortion, all relative representation alignment values (*ρ*) were roughly 0, indicating no consistent relationship between EC spaces and GloVe embeddings. As in the functional alignment experiments, as distortion decreases, *ρ* increases for VQ-VIB_𝒩_, until reaching English response levels. At that point, *ρ decreases*, reflecting a worsening alignment between the spaces as informativeness increases further. (Similar trends hold for VQ-VIB_𝒞_ and are plotted in Figure E.4, omitted here for clarity.) Thus, VQ-VIB models achieve peak relative representation alignment by matching the informativeness of the simulated English speaker. This is a key result in our experiments, highlighting the importance of matching information-theoretic properties of EC and natural language for the greatest alignment.

## EXPERIMENT 3: GENERALIZING TO 2D NAVIGATION

In our first two experiments, we showed how agents could learn to communicate in reference games, and how terms in the ICEC framework regulated important aspects of communication; in our final experiment, we showed how the same framework can be applied to training agents in (simulated) grounded environments. As before, we found that penalizing complexity led to simpler systems. At the same time, encouraging informativeness improved the convergence of communication to more meaningful protocols, suggesting an important pressure for language emergence.

### Experiment Setup

We developed a two-dimensional simulated world, depicted in Figure 4c. In the world, a speaker agent observed a target, spawned at a location generated uniformly at random in the map, while a listener agent only observed its own location in the map (but not the target location). Both agents achieved reward equal to the negative Euclidean distance from the listener to the target, so utility was maximized if the listener moved straight to the target. Given that the listener could not observe the target location, the speaker and listener had to jointly learn to use the environment’s communication channel, in which the speaker could broadcast communication at the first timestep in the environment, which the listener could observe. Thus, the optimal policy to maximize reward would consist of the speaker communicating about the location of the target. Agents were trained using a standard policy-gradient method (Multi-Agent Deep Deterministic Policy Gradient [MADDPG, Lowe et al. (2017)]), as well as the informativeness and complexity terms introduced in the ICEC framework.

We conducted two types of experiments in this domain by varying *λ_I_* or *λ_C_*. In our first experiment, we trained new teams of agents from scratch with *λ_U_* = 1.0 and *λ_C_* = 0.01 while varying *λ_I_* across trials. (Similar trends were observed for other small fixed values of *λ_C_* ∈ {0.001, 0.0001}.) This exposed how different informativeness pressures led to improved convergence during training. In our second experiment, we fixed *λ_U_* = 1.0 and *λ_I_* = 1.0 while slowly increasing *λ_C_* within a trial. This revealed how decreasing complexity led to different communication strategies.

### Results

We observed three main trends in our 2D Navigation experiments: 1) increasing *λ_I_* led to faster convergence to higher reward for all agent architectures, 2) increasing *λ_C_* led to less complex communication and coarser discretizations of the 2D space, and 3) VQ-VIB methods outperformed other agent architectures in achieving greater utility, for the same complexity, as other agents, likely due to the semantically-meaningful communication space that VQ-VIB agents learned.

**Figure 12.**
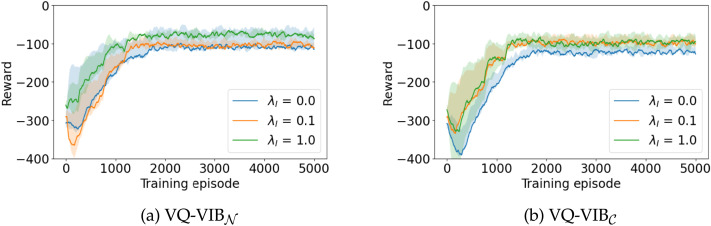
Training curves for VQ-VIB_𝒩_ and VQ-VIB_𝒞_. During training, mean reward increased for all methods, but increasing *λ_I_* (different curves) led to higher mean rewards.

As shown in Figure 12, greater *λ_I_* led to higher rewards. Each curve in Figure 12 represents the team utility (calculated as the average distance from the listener to the goal) over the course of training VQ-VIB_𝒩_ and VQ-VIB*_C_* teams with different *λ_I_* values, averaged over five trials. As shown for these VQ-VIB architectures, and for baselines architectures included in Appendix F, which showed similar trends, increasing *λ_I_* led to faster convergence to higher mean rewards. Given the very low *λ_C_* used during training, the performance differences for different *λ_I_* values indicate an important benefit of informativeness pressures in the shaping the training and emergence of communication among intelligent systems.

**Figure 13.**
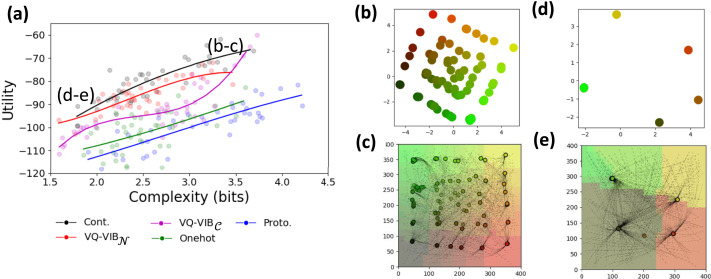
Varying complexity solutions in the 2D world environments. (a) Utility vs. complexity reflect task-agnostic tradeoffs between referring to specific target locations (high utility) and using less complex communication. Among discrete methods, VQ-VIB methods outperformed other architectures. Visualizations of VQ-VIB_𝒞_ agent traces at high (c) and low (e) complexity show how agents discretized the space into increasingly large regions. Visualization of the communication for these agents (b and d) show that communication vectors were embedded in a meaningful space: nearby tokens (in communication space) referred to nearby targets (in physical space). A video of communication evolution for VQ-VIB_𝒩_ shows smooth evolution between high and low complexity solutions.

In our next experiment, we trained five teams with *λ_U_* = 1.0, *λ_I_* = 1.0 until convergence (5,000 episodes) and then slowly increased *λ_C_* for an additional 10,000 episodes. This smoothly decreased the complexity of communication for all agents. Results from that experiment are shown in Figure 13.

Comparing across architectures, we found that both VQ-VIB models achieved greater utility, for the same complexity, than other methods. Figure 13a plots team utility for different architectures and random trials, recorded while annealing *λ_C_*. (The plotted complexity metric is calculated via the variational bound used during training, providing an upper bound on true complexity.) Increasing *λ_C_* led to less-complex communication (moving to the left along the *x* axis) and lower utility (moving down along the *y* axis). All agent architectures demonstrated the expected decrease in utility as complexity decreased, but some architectures (VQ-VIB) achieved greater utility than others for the same complexity value. The black curve for continuous communication represents a theoretical upper bound on utility, which the VQ-VIB agents nearly match despite being a discrete communication method.

Visualizations of the VQ-VIB_𝒞_ communication space, and discretizations of the 2D space, suggests that VQ-VIB agents outperformed other discrete methods due to their semantically-meaningful communication space (similar to in the reference games in prior experiments). Figure 13 (c and e) depict how VQ-VIB_𝒞_ agents discretized the 2D space at high and low communicative complexity values, respectively. Each background color represents the set of locations referred to by the same communication vector, and each point represents the mean location of the listener agent, based on that communication vector (with actual paths traced in black). Thus, Figure 13 shows that high-complexity communication led to fine-grained discretization of the continuous space, while low-complexity communication led to much cruder discretizations. This aligns with intuitions of humans modulating the complexity of spatial navigation from highly complex (e.g., GPS coordinates when navigating to a precise location) to crude (e.g., “North,” “South,” “East,” or “West” for high-level directions).

Visualizations of the VQ-VIB_𝒞_ communication space, included in Figure 13 (b and d), suggest that VQ-VIB agents achieved greater utility than other methods by communicating via a discrete set of symbols embedded in a semantically-meaningful space. Figures 13 (b and d) show the learned communication vectors at high and low complexity. (Agents were trained to communicate via 2D tokens to support direct visualization of the communication vectors.) Each token is visualized as a point in the communication space, colored by the mean location it referred. At both high and low complexity levels, the communication tokens clearly reflect a semantically-meaningful space: nearby tokens (in communication space) referred to nearby targets (in the simulated world). These results closely parallel the findings from our color and ManyNames reference games, wherein VQ-VIB agents learned semantically-meaningful embeddings, thus demonstrating consistent trends across domains.

## DISCUSSION

We have presented a framework for studying information-constrained emergent communication in artificial agents. Our framework integrates a fundamental information-theoretic tradeoff between complexity and informativeness, known as the Information Bottleneck (IB) principle, that has been shown to characterize human semantic systems across languages, with the principle of task-specific utility maximization in game-theoretic approaches to language evolution. To efficiently train agents with respect to the generalized tradeoff between utility, informativeness, and complexity, we proposed a novel deep-learning method called Vector Quantized Information Bottleneck (VQ-VIB). In addition to enabling a flexible parametrization of the IB encoder and decoder, a key feature of this method is that it allows agent to develop communication systems in which discrete signals are embedded in a continuous conceptual space, analogous to the notion of word embeddings in natural language processing.

Across our experiments, two trends have emerged: (1) controlling complexity and informativeness, within the ICEC framework, enabled faster learning, greater generalization, and more human-like communication than previously proposed emergent communication methods, and (2) the VQ-VIB method is particularly adept at learning semantically-meaningful complexity-limited communication. More specifically, in all experiments and for all neural network architectures, we found that increasing pressure for informativeness (by increasing *λ_I_*) tended to induce faster convergence. Our results in the ManyNames domain and 2D world further demonstrate that pressure for informativeness facilitates greater communicative generalization and open-domain communication. Simultaneously, limiting the complexity of communication (by increasing *λ_C_*) was important for aspects of human-like communication, as shown via comparisons to color-naming systems and natural language word embeddings. Beyond establishing general characteristics of terms in our ICEC framework, we found that across all our experiments, VQ-VIB outperformed other EC methods by achieving greater utility for the same level of complexity. Our qualitative evaluation of the emergent systems suggest that this boost in performance is likely due to the semantically-meaningful communication embedding space afforded by VQ-VIB. Unlike onehot agents, for which all communication vectors were equidistant and orthogonal, VQ-VIB agents embedded discrete communication signal in a continuous space and, through visualization in all experimental domains, we found that this space encoded important perceptually- and conceptually-grounded semantic properties of the inputs. We also believe that this grounded embedding space in VQ-VIB enables out-of-domain generalization as evident our the ManyNames study.

Our findings raise several important questions for future work to further advance our understanding of how communicative pressures are instantiated in the real world. For example, while utility may be reasonably modeled via the success of actions in the world, it is unclear *a priori* how human speakers and listeners could estimate the informativeness of communication without direct access to others’ mental states. New research inspired by Theory of Mind (ToM) capabilities could shed important light on such questions. Another promising direction for future work would be to study the relationship between multi-task utility and informativeness. While we trained emergent communication agents with a single utility function, one might also consider a multi-task framework in which agents must accomplish many rewards simultaneously.

More broadly, our work demonstrates how combining cognitively-motivated optimality principles with state-of-the-art deep learning tools could benefit both AI and cognitive science. From an AI perspective, our approach has yielded AI agents with improved capabilities for developing human-like communication systems on their own, without any human supervision. From a cognitive science perspective, our theoretical framework provides a potential mechanistic agent-based account for how a near-optimal lexicon, as seen across languages, may evolve. We also believe our framework could be further used to test how various social and environmental factors may shape language and its evolution.

## ACKNOWLEDGMENTS

NZ and MT thank MIT, at which they did much of the work presented in this paper. RPL gratefully acknowledges support from the MIT Quest for Intelligence and the Simons Center for the Social Brain at MIT.

## AUTHOR CONTRIBUTIONS

NZ, MT, RPL, and JS conceived of the research; MT and NZ designed the experiments with input from RPL and JS; MT implemented models, conducted numerical experiments and analysis, and created visualizations, with assistance and guidance from NZ; MT and NZ analyzed and interpreted the results; MT and NZ wrote the paper with input from RPL and JS.

## DATA AVAILABILITY STATEMENT

Our main code is available at https://github.com/mycal-tucker/opmi-vqvib and https://github.com/mycal-tucker/vqvib_neurips2022. IB analysis and the IB color naming model is available at https://github.com/nogazs/ib-color-naming. The ManyNames dataset is available at https://github.com/amore-upf/manynames and the World Color Survey dataset is available at https://linguistics.berkeley.edu/wcs/data.html.

## Notes

^1^ Zaslavsky et al. (2018) use the term “accuracy” where we use the term “informativeness;” in some of our experiments in which we train agents, there is a notion of team accuracy that is measured by a utility function, so “informativeness” better illustrates the distinction between these terms.^2^ Traditional Markov Decision Process (MDP) frameworks express similar notions of states *S*, actions *A*, and reward functions *R*:*S* × *A* → ℝ, which parallel *X, Y*, and *U*(*X*, *Y*), respectively (Sutton & Barto, 2018). We use our notation to highlight similarities with classification tasks, where *y* denotes a prediction or decision.^3^ For completeness, we repeated these experiments using a REINFORCE training mechanism in which gradients were not passed between the listener and speaker. Results were largely unchanged; details are included in Appendix D.^4^ In our experiments, onehot agents never converged to greater than 50% utility for *λ_C_* = 0.01, as was used for other models. We therefore set *λ_C_* = 0.0 for onehot, which biased communication to have higher utility and informativeness, for the same *λ_I_* and *λ_U_*.

## Figures and Tables

**Figure A.1:**
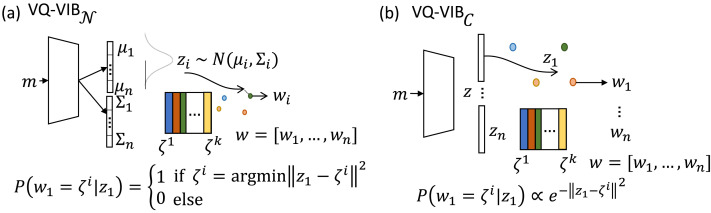
VQ-VIB_𝒩_ (a) and VQ-VIB_𝒞_ (b) speaker architectures for combinatorial codebooks.

**Figure D.1:**
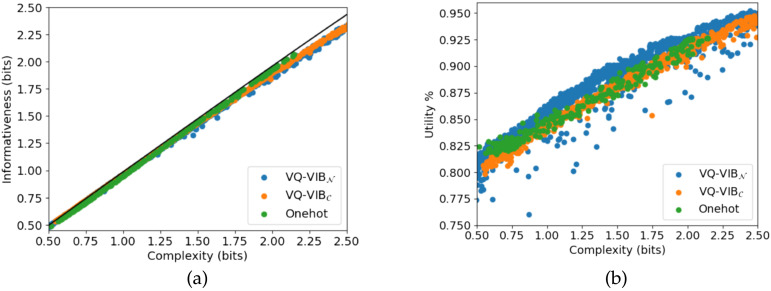
Informativeness vs. complexity (a) and utility vs. complexity (b) in the color reference game for models trained with REINFORCE. We observed similar trends as when training via backpropagation: all models were similarly IB-optimal (a), and VQ-VIB_𝒩_ tended to achieve higher utility, for the same complexity, as other models.

**Figure E.2:**
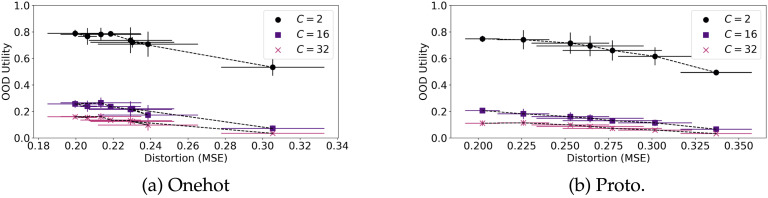
OOD accuracy vs. distortion for onehot and Proto. architectures, and varying *C*. As in Figure E.1 for VQ-VIB models, decreasing distortion was associated with increased OOD utility.

**Figure E.3:**
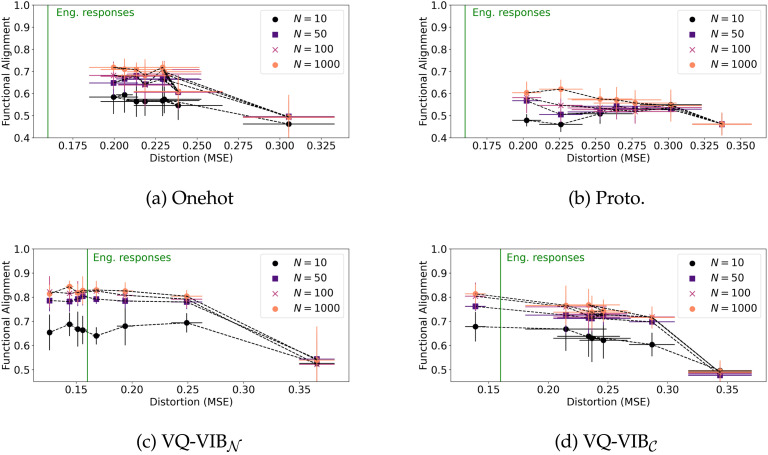
Functional alignment for different architectures for varying amounts of alignment data, *N*. Increasing *N* improved alignment slightly, but performance typically plateaued after reaching English response informativeness.

**Figure E.5:**
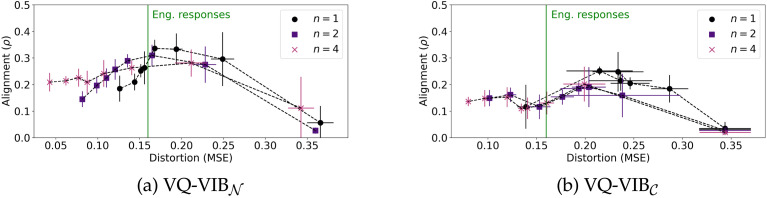
Relative representation alignment for VQ-VIB_𝒩_ and VQ-VIB_𝒞_, for varying combinatorial token settings (*n*). Increasing *n* decreased the distortion for both model types (note the changed *x* axis scale) but worsened alignment.

**Figure F.1:**
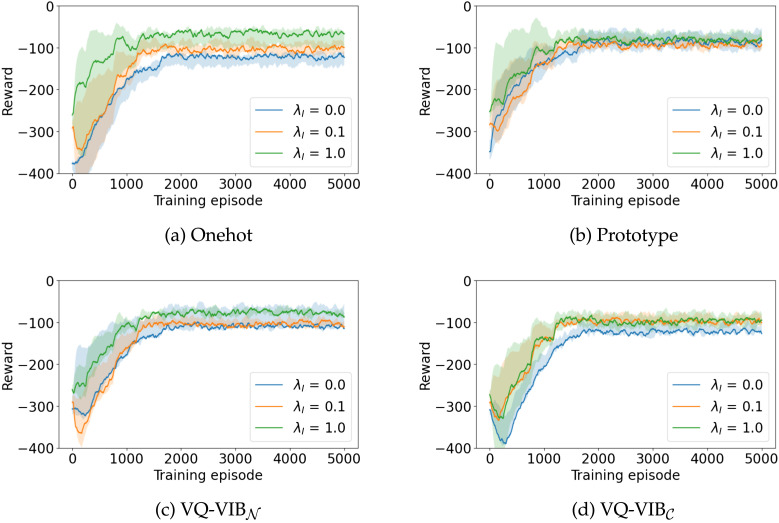
Team utility as a function of training episode for different model architectures (different plots) and different *λ_I_* values (different curves). Increasing *λ_I_* improved performance for all model types.

## A: COMBINATORIAL CODEBOOK

In the main paper, we propose two VQ-VIB architectures, VQ-VIB_𝒩_ and VQ-VIB_𝒞_, both of which use a learnable codebook to discretize communication. In this section, we show how simple changes to the neural network architectures support dramatic increases in the codebook sizes while retaining support for the variational bounds on complexity from the original architectures. Diagrams of both updates architectures are included in Figure A.1.

In VQ-VIB_𝒩_, a feedforward encoder outputs [*μ*_1_, …, *μ_n_*] and [Σ_1_, …, Σ*_n_*] for *μ_i_*, Σ*_i_* ∈ ℝ^*d*/*n*^. That is, rather than producing the mean and variance for a single *d*−dimensional normal distribution, the encoder outputs the parameters for *n* normal distributions, each in ℝ^*d*/*n*^. The learnable codebook, =*ζ_i_* ∈ ℝ^*d*/*n*^; *i* ∈ [1, *K*] is similarly updated so that each codebook element is in ℝ^*d*/*n*^. Thus, each $z_{i}∼N\left(\mu_{i},\Sigma_{i}\right)$ is discretized via the standard discretization process of snapping to the nearest element of the codebook. Lastly, the overall discrete communication is generated by concatenating all discrete subtokens, generating a final discrete communication vector *w* ∈ ℝ*^d^*

In VQ-VIB_𝒞_, the feedforward encoder and codebook are similarly updated to generate *n*, $\frac{d}{n}-$dimensional discrete representations that are concatenated together. As shown in Figure A.1b, the encoder generates *n* continuous representations: *z*_*i*_ ∈ ℝ^*d*/*n*^, *i* ∈ [1, *n*]. As in VQ-VIB_𝒩_, the learnable codebook defines *K* vectors: = *ζ_i_* ∈ ℝ^*d*/*n*^; *i* ∈ [1, *K*]. Each *z*_*i*_ is discretized using the VQ-VIB_𝒞_ sampling process based on negative distance from *z*_*i*_ to each element of the codebook. Lastly, these discrete representations are concatenated into *w* ∈ ℝ*^d^*.

Conceptually, updating VQ-VIB_𝒩_ and VQ-VIB_𝒞_ to support concatenations of discrete tokens only differs from the prior architecture by expanding the finite set of possible communication vectors. The architectures presented in the main paper may be thought of as a special case of this more general combinatorial setup by setting *n* = 1. By increasing *n*, however, one can dramatically increase the effective codebook size of agents: e.g., for *K* = 1024, as used in our ManyNames experiments, *n* = 1 generates 1024 discrete representations, whereas *n* = 4 generates 1024^4^ ≈ 10^12^ representations, without increasing the number of weights in the neural nets. This increased codebook size in turn supported more complex and informative representations which in some cases improved model performance (e.g., for OOD generalization) but in others was unnecessary (e.g., maximum representation alignment was achieved at low complexity, so increasing *n* did not help alignment).

## B: PENALIZING ENTROPY

In the main paper, we defined the VQ-VIB training objective in Equation 12, which included a tie-breaking entropy loss. Here, we explain the motivation behind this term and how it was calculated for VQ-VIB_𝒩_ and VQ-VIB_𝒞_ models.

Prior art shows that, for any given complexity level, there are many possible IB-optimal solutions with different effective codebook sizes *k* = ∣ {*ζ_i_* ∈ *ζ*: ∑*_m_*ℙ(*m*)*q*(*ζ_i_*|*m*) > 0∣ (Zaslavsky, 2020). This measurement of effective codebook size captures how many distinct tokens are used by a speaker. Intuitively, for the same complexity level, a speaker may use relatively few tokens (each token encoding a quite distinct meaning), or use many different tokens that are sampled quite stochastically (e.g., two tokens might encode the same meaning, and the speaker randomly chooses between the tokens).

We were interested in EC systems with the minimal *k*, for a given complexity level, as they encode the same amount of information with the smallest effective codebook size. Therefore, to bias EC agents towards small *k*, we wished to penalize the entropy of the categorical distribution over tokens, breaking the tie within a complexity equivalence class to favor small-codebook solutions.

For VQ-VIB_𝒞_, we could directly measure and penalize the entropy of the categorical distribution, as it was calculated while sampling tokens: ℙ(*ζ_i_*|*m*) ∝ *e*^‖*ζ_i_*−*z*(*m*)‖^2^^. VQ-VIB_𝒩_, however, does not support exact calculation of the categorical distribution over tokens. We therefore approximated the categorical distribution by using the same probability function as for VQ-VIB_𝒞_: proportional to *e*^‖*ζ_i_*−*z*(*m*)‖^2^^.

As shown in Equation 12, we weighted the entropy loss term by a small scalar value: *ε* * *λ_C_*. Given that we merely sought to penalize entropy within a complexity equivalence class, rather than use this term directly to control agents, we typically set *ε* to small values: in the 2D world environments, *ε* = 0.01, and in the color and ManyNames domains, *ε* = 0.05. These values were chosen by training teams to low complexity solutions and evaluating how many tokens were used. *ε* was increased until the number of tokens shrank to roughly log_2_ of the complexity.

## C: BASELINE ARCHITECTURES

Here, we elaborate upon some of the neural network architectures we used in experiments other than the VQ-VIB methods.

The two discrete EC architectures we started with were onehot and Proto.. As discussed in the main text, onehot agents mapped inputs to a onehot vector. Proto. agents, introduced by Tucker et al. (2021), internally compute a onehot vector, which is then multiplied by a “prototype matrix” to generate discrete representations in a continuous space. While both methods generate discrete communication, neither supports variational bounds on complexity. Thus, we adapted these methods to generate strengthened baselines that fit within the ICEC framework.

Our updated onehot baseline used a variational bound on the categorical distribution over the communication dimension. As in VQ-VIB_𝒞_, onehot agents generated a categorical distribution over which token to emit and sampled from this distribution using the gumbel-softmax trick (Maddison et al., 2017). We therefore similarly bounded complexity via $I\left(m;z\right)\leq E\left[D_{KL}\left[q_{\theta }\left(w|m\right)∥r\left(w\right)\right]\right]$ where we set *r*(*w*) to a uniform categorical distribution.

Our updated Proto. baseline used a variational bound on complexity based on normal distributions. Variational Proto. agents used a standard Proto. speaker, which generates a discrete representation, *z*, in a continuous space. Agents also produced Σ, representing the variance of a normal distribution. Lastly, the Proto. agent’s communication was generated by sampling from a normal, centered at the discrete representation, with variance Σ: $w∼N\left(z,\Sigma \right)$. Thus, Proto. agent communication passed through a discrete bottleneck but was ultimately continuous. Given this sampling mechanism, we used the variational bound on complexity often used in VAEs: $I\left(m;w\right)\leq E\left[D_{KL}\left[q_{\theta }\left(w|m\right)∥r\left(w\right)\right]\right]$, which is tractable for a unit normal prior, *r*(*w*).

## D: COLOR REFERENCE GAME FURTHER RESULTS

In our main color reference game experiments, we trained agents via a supervision loss, which we backpropagated through the speaker and listener.

Here, we briefly present results from a similar setup but with a different training method: REINFORCE (Williams, 1992) Using this reinforcement-based training mechanism, the speaker was only indirectly trained to maximize team accuracy, without the ability to backpropagate through the listener. Prior art in EC has employed both REINFORCE and supervisory methods for reference games (including, in particular, color) and found that training with REINFORCE was less stable and led to communication with lower communicative complexity (Chaabouni, Kharitonov, et al., 2021).

Results from our agents, trained with REINFORCE, are depicted in Figure D.1. As before, we found that all models achieved near optimal informativeness and that VQ-VIB_norm_ achieved greater utility than other methods (for a given complexity level).

Furthermore, we note that training with positive *λ_I_* consistently induced more complex communication than cases with *λ_I_* = 0, as done in prior art. Chaabouni, Kharitonov, et al. (2021) trained 180 teams from scratch to overcome random failures of model training; in our results we trained 5 teams from scratch and each team converged to useful communication. In addition, whereas the 180 teams trained by Chaabouni, Kharitonov, et al. (2021) never learned communication more complex than 2.4 bits, we found that VQ-VIB models consistently surpassed 2.5 bits.

Thus, we found that the results in our main paper generalized to different training mechanisms and, moreover, the ICEC framework appeared capable of addressing important limitations identified in earlier work.

Lastly, when training agents with a supervision loss (not REINFORCE), we found, yet again, the importance of the ICEC framework in generating human-like naming systems. We compared to onehot and VQ-VAE agents trained with only utility rather than the full ICEC framework (*λ_I_* = *λ_C_* = 0). Results for such agents are depicted in Figure D.2.

Each point in Figure D.2 corresponds to the behavior for an agent with a hardcoded codebook size from 2 to 10. By varying the codebook size, we could indirectly control the resulting complexity of communication. This indirect control, however, affords less finegrained control than our ICEC framework, and is cognitively less plausible.

Therefore, in our final set of color experiments, we trained a variational version of onehot communication. Training within the ICEC framework, we reproduced important behaviors of smoothly modifying the complexity of communication. Metrics of efficiency and similarity to human languages for this improved onehot baseline, as well as VQ-VIB_𝒞_ (and results already reported in the main paper) are included in Table D.1. We note that, even as the Onehot - Variational efficiency metrics are similar to VQ-VIB models, VQ-VIB_𝒩_ continued to achieve greater utility than onehot, due to the semantically-meaningful communication space.

**Table D.1:** Mean (standard deviation) evaluation over five trials of artificial color communication systems, compared to human naming data and an IB-optimal bound. The three variational methods (VQ-VIB_𝒩_, VQ-VIB_𝒞_, and Onehot - Variational), trained in our ICEC framework were closer to human languages (lower gNID - generalization of Normalized Information Distance) and closer to the IB bound (lower efficiency loss) than utility-only methods (Onehot and VQ-VAE).

	VQ-VIB_𝒩_	VQ-VIB_𝒞_	Onehot - Variational	Onehot	VQ-VAE
Human-agent gNID	0.151 (0.00)	0.153 (0.01)	0.154 (0.00)	0.42 (0.16)	0.38 (0.15)
Efficiency loss	0.024 (0.00)	0.022 (0.00)	0.023 (0.01)	0.06 (0.01)	0.08 (0.01)

## E: MANYNAMES FURTHER RESULTS

In the main paper, we included OOD utility, functional alignment, and relative representation alignment results for some of the models in the ManyNames reference game. Here, we include results from varying *n* and varying *C* at test time.

### Generalization

Trends from our main generalization experiments indicated that increasing *λ_I_* and informativeness tended to enable greater generalization performance across models. Here, we examine such trends as we varied important task and model parameters.

First, by varying the number of candidate images used at test time, *C*, we exposed important differences between model capabilities. Chaabouni, Strub, et al. (2021) found that such analysis was important for uncovering differences in generalization capabilities. Therefore, we tested agents with *C* ∈ [2, 16, 32]; note, however, that all models were trained with *C* = 2. Thus, increasing *C* tested a different sort of generalization to a harder task setting. Second, we evaluated VQ-VIB models for *n* ∈ [1,2,4]. Recall that *n* is the combinatorial parameter that set how many codebook elements to concatenate into a single message.

OOD generalization results, for varying *C* and *n*, are included in Figure E.1. As expected, increasing *C* decreased utility, as the task became substantially more challenging. Notably, for all *C*, the trend from the main paper of lower distortion supporting greater utility continued to hold.

Examining results for *n* > 1, we see that this trend held even as distortion continued to decrease further. Increasing *n* clearly decreased distortion (note how curves shifted to the left along the *x* axis as *n* increased) which in turn was associated with greater utility. These improvements are particularly notable in comparison to onehot or Proto. performance, plotted in Figure E.2. For example, for *C* = 32, VQ-VIB_𝒩_ achieves up to 60% accuracy for *n* = 4, whereas onehot and Proto. accuracy peaks at approximately 17% and 13%, respectively.

Overall, we found a strong trend between informativeness and OOD utility across architectures. This trend also explains, to a large degree, inter-architecture differences: VQ-VIB models tended to achieve lower distortion, which in turn led to greater utility.

### Functional Alignment Analysis

In some of our ManyNames experiments, we trained a linear model to map from GloVe embeddings to EC vectors. In the main paper, we reported results based on fitting this model with *N* = 100 randomly-selected images and associated labels. In Figure E.3, we present results for different *N*.

As expected, increasing *N* increases translation performance, but only up to a point. More importantly, regardless of *N*, all plots exhibit similar plateauing behavior: at high distortion (low informativeness), performance improved as distortion decreased, but only up to the estimated English informativeness level, at which point performance roughly plateaued. Lastly, VQ-VIB architectures tended to outperform the other architectures, both by achieving greater alignment for the same distortion and by reaching lower distortion values.

### Relative Representation Alignment

Just as we conducted further analysis for functional alignment in the previous section, in this section we examined relative representation alignment trends for different neural architectures. In the main paper, we found that alignment peaked when EC distortion roughly matched English distortion, and that VQ-VIB_𝒩_ models tended to achieve greater alignment than other models.

First, complementing Figure 10, we plotted relative representation alignment for all model architectures, including VQ-VIB_𝒞_ in Figure E.4. Notably, both VQ-VIB methods achieve greater alignment than other models, and both achieve greatest alignment near the estimated distortion from English responses.

Next, we examined alignment for both VQ-VIB models for varying *n*, the parameter specifying how to combine codebook elements into discrete communication. Results are plotted in Figure E.5.

Two main trends are apparent in Figure E.5. First, as identified in our OOD experiments for varying *n*, increasing *n* led to lower distortion (higher informativeness). Second, and most importantly, despite the increased informativeness, increasing *n* tended to result in *lower* relative representation alignment. This reinforces the importance of setting the “right” informativeness to match the English data, and that simple architectures can, at least in this domain, learn the most human-like representation space.

## F: TRAINING CURVES

In Figure 12, we showed training curves in the 2D simulated word for VQ-VIB_𝒩_ and VQ-VIB_𝒞_ agents. Increasing *λ_I_*, the pressure for informativeness, caused both model types to converge faster and to a higher mean reward. For completeness, we include similar training curve results for onehot and Proto. agents in Figure F.1.

As before, we found that, for all architecture types, increasing *λ_I_* continued to induce higher-reward policies earlier in training. This indicates that informativeness can be a powerful indirect pressure towards high-utility policies, especially in multi-agent reinforcement learning settings, which are notoriously hard to train models in (Eccles et al., 2019).
